# Nanocarrier‐Based Systems for Targeted Delivery: Current Challenges and Future Directions

**DOI:** 10.1002/mco2.70337

**Published:** 2025-08-21

**Authors:** Zichen Xu, Yongyi Xie, Wenjie Chen, Wei Deng

**Affiliations:** ^1^ School of Biomedical Engineering University of Technology Sydney Ultimo NSW Australia; ^2^ Guangdong Province & NMPA & State Key Laboratory School of Pharmaceutical Sciences Department of Emergency The Second Affiliated Hospital Guangzhou Medical University Guangzhou China

**Keywords:** active targeting, drug and gene delivery, nanocarrier, nuclear localization signals, nuclear targeting, targeted delivery

## Abstract

Nanomaterials have become promising platforms in the field of drug and gene delivery, offering unique advantages over traditional therapeutic approaches. Their tunable physicochemical properties enable improved pharmacokinetics and therapeutic performance. A wide range of nanocarriers, including lipid‐based, polymer‐based, and hybrid systems, have been rapidly developed and are attracting increasing attention in both preclinical and clinical research. However, despite promising preclinical outcomes, these systems still encounter critical challenges in achieving precise delivery to specific tissues, cells, and intracellular compartments. This review provides a comprehensive assessment of recent advances in the design and application of nanocarriers for targeted delivery, with emphasis on strategies designed for nuclear targeting. In the context of nuclear targeting, it explores passive approaches involving modulation of particle size, morphology, and surface charge, alongside active targeting strategies incorporating nuclear localization signals and other ligands. In addition to highlighting progress, the review examines the limitations associated with delivery efficiency, off‐target effects, and barriers to clinical translation. By addressing both advances and ongoing challenges, this review provides valuable insights into the design and engineering of targeted nanocarriers. These developments are crucial for unlocking the full potential of precision nanomedicine.

## Graphical abstract

Recent advances in nanocarrier‐mediated delivery for nuclear targeting

- *Cell nucleus*: key target for improved therapeutic outcomes including chemotherapy, photodynamic therapy, and gene therapy
- *Nuclear‐targeted drug delivery strategy*: including nuclear localization sequence (NLS)‐mediated active targeting and passive strategies
• Challenges in nuclear‐targeted delivery systems

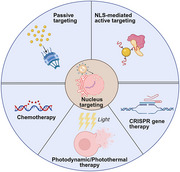

## 1 Introduction

Nanocarriers have been widely developed and applied to various biomedical applications primarily due to their ability to improve the pharmacokinetic profiles of drugs and protect them from enzymatic degradation [1]. However, their therapeutic effectiveness limited as only a small portion of administered nanocarriers reach the intended target tissues and cells [2]. Studies have shown that a mere 0.7% of nanomaterial drugs achieved successful accumulation near tumors while most are taken by normal tissues, leading to off‐target toxicity and unwanted side effects [3]. Furthermore, many therapeutic targets are located within specific subcellular organelles [4]. These facts highlight the pressing need for the development of effective targeted delivery.

To enhance the precision and efficiency of nanocarrier‐based therapies, various strategies have been developed for tissue‐ and cell‐specific targeted delivery, which are broadly classified into passive and active targeting approaches. While these strategies have significantly improved the accuracy and efficacy of drug delivery, they still face critical limitations. Specifically, once therapeutic agents reach the target cells, they often fail to localize precisely within specific subcellular organelles such as mitochondria or the nucleus [5]. This lack of intracellular specificity can compromise therapeutic effectiveness. Consequently, there is a growing emphasis on shifting from tissue‐ and cell‐level targeting toward more refined subcellular‐level targeting. Such precision enables drugs to directly interact with key intracellular structures and molecular pathways, thereby substantially enhancing therapeutic outcomes [6].

The term “subcellular” refers to the level of organization within a cell that is smaller than the entire cell. It includes various structures and compartments, each having an important function [7]. Nucleus has been widely investigated as a subcellular organelle. Precise target of the nucleus allows for the selective delivery of therapeutic drugs, enhancing the therapeutic efficacy of treatments [8, 9]. However, nuclear targeting is a challenging journey that encounters various obstacles while attempting to reach the intended destination. These obstacles mainly include clearance by the phagocyte system [10], biological barriers (e.g., the blood–brain barrier [BBB]) [11], and endo/lysosomal degradation [12, 13, 14].

Over the past decade, nuclear‐targeted delivery has remained a prominent research focus. Most studies have focused on specific types of nanocarriers, such as gold and chitosan (CS)‐based nanoparticles, as well as on certain application areas in cancers. However, both the number of publications and citation rates in this field appear to have reached a plateau over the past 4 years. Some bottleneck issues may need to be cracked urgently to advance the development of this fundamental research area, facilitating the continuous progress. A deeper investigation into publications identified 12 review articles, yet most of these primarily address drug delivery for cancer therapy. The existing review paper primarily focuses on the subject of gene delivery to the nucleus [15], which represents a relatively narrow aspect within this field.

In order to address this gap, our review aims to provide an updated overview of advancements in nuclear‐targeted strategies used for both drug and gene delivery systems. We begin by summarizing the progress made in the development of targeted delivery systems, focusing on lipid‐, polymer‐, and metal‐based nanocarriers and their applications across various disease models. This is followed by a brief overview of key subcellular organelles and their functions, laying the groundwork for understanding the importance of directing therapeutic agents to specific intracellular compartments, particularly the nucleus. We then focus on the advancements that have been made in the development and utilization of nuclear‐targeted strategies. This section particularly discusses the use of nuclear localization signals to specifically deliver therapeutic agents to the nucleus. Last, we identify the potential challenges associated with targeted nanocarriers and outline future directions for advancing precision nanomedicine by utilizing targeted delivery systems.

## 2 Types of Nanocarriers for Targeted Delivery

Over the past decades, nanoparticle‐based drug delivery systems have been widely used for the targeted delivery of therapeutic agents, reducing the nonspecific side effects associated with these drugs [16, 17, 18, 19, 20]. Various types of nanocarriers, including liposomes, polymeric nanoparticles, metal nanoparticles, and hybrid nanocarriers, have been developed for drug and gene delivery [21, 22]. Each type has unique characteristics in delivery applications, depending on its composition and functionality.

### 2.1 Liposomes

Liposomes are among the most extensively used drug delivery systems due to their biocompatibility and safety [23, 24]. Their amphiphilic structure allows for the simultaneous loading of hydrophilic drugs in the aqueous compartment and hydrophobic molecules in the lipid bilayer [25, 26]. The most commonly utilized lipids in the formulation include phospholipids and cholesterol [27]. These lipids offer well‐established safety and biocompatibility, and their structural diversity enables tailored physicochemical properties, making them highly suitable for applications across pharmaceuticals, cosmetics, and nutraceuticals. A well‐known example is Doxil, a clinically approved doxorubicin (DOX)‐loaded liposome used in cancer therapy. Compared with free drugs, the liposomal formulation reduced systemic toxicity while enhancing tumor accumulation via the enhanced permeability and retention (EPR) effect [28, 29]. Liposomes are also utilized for gene delivery by serving as carriers. For example, plasmid DNA can be mixed with lipid solutions to form a liposomal–DNA complex, which can fuse with the cell membranes of various cell types, enabling efficient gene transfer [30, 31]. Despite their advantages, liposomes still face challenges such as drug leakage and limited targeting specificity, requesting additional surface modifications to enhance circulation time and improve tissue‐specific accumulation [24, 32]. In addition, liposomes are inherently unstable in the bloodstream. They rapidly adsorb serum proteins and are cleared by hepatic macrophages, limiting their stability and circulation time [27, 33, 34]. While PEGylation is widely used to modify liposome surfaces and extend circulation time, it can induce immune responses (e.g., anti‐PEG antibodies), thereby reducing efficacy after repeated dosing [35]. Recent advancements in liposome targeting capabilities have primarily focused on stimuli‐responsive designs and modifications to lipid composition [36, 37]. For example, Enzian et al. [38] designed a photosensitive bilayer liposomal system composed of 1,2‐dipalmitoyl‐sn‐glycero‐3‐phosphocholine and 1,2‐distearoyl‐sn‐glycero‐3‐phosphoethanolamine‐N‐[poly(ethylene glycol)‐2000]. The liposomes encapsulated three distinct photosensitizers: porphyrin 5,10‐di(4‐hydroxyphenyl)‐15,20‐diphenyl‐21,23H‐porphyrin (5,10‐DiOH), its chlorinated derivative (5,10‐DiOH‐chlorin), and bacteriochlorin (5,10‐DiOH‐bacteriochlorin), each of which facilitated efficient drug release under irradiation with different wavelengths of light [38]. Upon light exposure, the system generated reactive oxygen species (ROS), inducing lipid degradation in the liposomal membrane and triggering controlled drug release. This strategy enables spatial and temporal control over drug delivery, with payload release occurring precisely at the target site upon external photoactivation. Yaroslavov et al. [39] developed a pH‐sensitive bilayer liposomal vesicle composed of egg phosphatidylcholine and a pH‐sensitive “activator,” which is a derivative of lithocholic acid bearing anionic and cationic groups at opposite ends of the steroid core. This system exhibited rapid drug release, with approximately 50–60% of the encapsulated anticancer agents released within minutes upon acidification [39]. pH‐sensitive liposomes exploit the acidic tumor microenvironment to destabilize the lipid bilayer structure and trigger localized drug release [39].

### 2.2 Polymeric Nanocarriers

Polymeric nanocarriers are composed of one or more types of synthetic or natural polymer units covalently bonded to achieve adjustable drug release profiles and multifunctional designs. They also offer simple formulations, such as poly (lactic‐co‐glycolic acid) (PLGA) [40]. Compared with liposomes, polymers exhibit greater stability and higher cargo retention efficiency [40, 41, 42]. This is due to their composition of block copolymers, in which the covalent bonds between blocks are more resistant to degradation than the ester bonds in phospholipids [43]. Additionally, the degradation of some polymer repeating units has a lesser impact compared with low‐molecular‐weight lipids [43, 44], and studies have shown that polymers can remain stable for up to 6 months at room temperature [45]. Furthermore, the high permeability of liposomes is attributed to their high lateral fluidity, a result of their low molecular weight, which leads to poor cargo retention efficiency [46, 47]. In contrast, polymers are more thermodynamically stable, with significantly lower lateral diffusivity, allowing them to retain cargo more effectively [43, 48].

Polymer carriers play an important role in gene delivery due to their ease of synthesis and adaptable properties [49]. For example, cationic polymers form stable complexes with genetic material through electrostatic interactions at physiological pH, protecting genes from degradation and facilitating cellular uptake via endocytosis [50]. These carriers exhibit high transfection efficiency as nonviral gene delivery systems. For example, Wan et al. [51] developed a supramolecular polymer system incorporating detachable diguanide ligands for the delivery of Cas9 ribonucleoprotein complexes targeting mutant *KRAS* genes. Compared with noncarrier systems, this platform demonstrated improved stability and significantly enhanced transfection efficiency [51]. In another study, Li et al. [52] designed dual‐grafted trimethyl CS nanoparticles modified with folic acid (FA) and 2‐(diisopropylamino) ethyl methacrylate for the codelivery of DOX and CRISPR/Cas9 plasmids. This formulation achieved a marked tumor regression rate of 91.0% in 4T1 tumor‐bearing mice [52].

However, the potential toxicity associated with polymeric nanoparticles remains a major challenge [53]. In addition, the structural complexity of these nanoparticles presents significant barriers to large‐scale manufacturing, necessitating rigorous characterization protocols to ensure batch‐to‐batch consistency and safety [54, 55]. Despite these limitations, clinically approved polymers represent meaningful progress and continue to hold strong potential for advancing the field of nanomedicine [56].

### 2.3 Metallic Nanocarriers

Metallic nanocarriers have been extensively studied for drug and gene delivery due to their unique physicochemical properties. Their small size and stability enable efficient binding with a wide range of cargo [57, 58]. In particular, gold nanoparticles (AuNPs) stand out as highly functionalized metallic carriers [59, 60]. For example, Lee et al. [61] developed a CRISPR–AuNPs vehicle for the direct delivery of Cas9 RNP and donor DNA in vivo via local administration. This system was used to correct gene alterations in an Ai9 mouse model of Duchenne muscular dystrophy [61]. Although AuNPs are generally regarded as biocompatible, concerns remain regarding their long‐term toxicity and potential accumulation in the body [62].

Metal‐organic frameworks (MOFs) are porous materials with high surface area, abundant pores, and tunable microenvironments [63]. These properties enable MOFs to enhance drug loading capacity and achieve controlled release at targeted lesion sites [64]. MOFs can also be used for gene delivery by loading various genetic cargo such as small interfering RNA (siRNA), messenger RNA (mRNA), and DNA [65, 66]. For instance, Zhou et al. [67] developed a mesh MOFs for intracellular delivery of DNAzyme in human immune cells, effectively inhibiting the expression of the EGR‐1 gene. Pore encapsulation is a technique that integrates functional molecules within the pores of nanocarriers and is commonly used for loading therapeutic cargos into MOFs [68]. However, molecules incorporated through surface adsorption and pore encapsulation often experience gradual leakage due to weak interaction forces [64]. Covalent binding offers a solution by forming strong covalent bonds with reactive groups on the target, ensuring more stable interactions [69]. However, this approach requires complex synthetic procedures and may alter the activity of therapeutic molecules [70]. While MOFs show great promise for drug delivery applications, further research is needed to fully understand their in vivo toxicity, degradation mechanisms, and pharmacokinetics [70].

### 2.4 Hybrid Nanocarriers

Numerous studies have demonstrated that the hybridization of two or more nanocarrier systems can lead to the formation of novel architectures capable of integrating the complementary advantages of each component, enhancing the delivery efficiency of therapeutic agents in cancer treatments [71]. Among these, lipid–polymer hybrid nanoparticles have shown particularly promising performance in gene and drug delivery, exhibiting superior intracellular delivery and transfection efficiency compared with their single‐component counterparts [72]. In this design, the lipid component enhances biocompatibility and cellular uptake, while the polymer contributes to improved structural stability, controlled drug release, and tunable physicochemical properties [73]. For instance, Sedef et al. [74] developed a hybrid delivery system composed of a lipid core made from a beeswax–olive oil mixture and a biopolymer shell made from BSA/dextran Maillard conjugates for the delivery of the highly lipophilic anticancer drug paclitaxel. This hybrid design exhibited optimized characteristics, including small particle size, pH stability, and efficient drug encapsulation. In addition, inorganic–polymer hybrid nanocarriers also hold significant therapeutic promise. For example, Mehrab et al. developed a composite nanocarrier made of graphene oxide, polyvinylpyrrolidone, and sodium alginate, which significantly improved the delivery and therapeutic effectiveness of 5‐fluorouracil (5‐FU) against colorectal cancer cells [75]. This system offers controlled and sustained release of 5‐FU at the cellular level, with the potential to improve therapeutic outcomes while minimizing the systemic toxicity commonly associated with traditional chemotherapy. Despite their multifunctional potential, hybrid nanocarriers still face several translational barriers, including complex fabrication processes, structural instability, potential toxicity, and regulatory uncertainty, all of which hinder their clinical implementation [76].

## 3 Mechanisms of Targeted Delivery

### 3.1 Passive Targeting

Passive targeting strategies are predominantly based on the EPR effect, a phenomenon unique to solid tumors [77, 78]. This effect arises from the increased permeability of tumor vasculature compared with normal tissues [77, 79]. In tumors, the endothelial cell gaps range from 100 to 780 nm, depending on the cancer type, whereas in healthy tissues, these gaps are significantly smaller, approximately 5–10 nm [80]. Consequently, nanocarriers within an optimal size range can preferentially extravasate into tumor sites. This mechanism has been successfully exploited in clinical applications, as demonstrated by the approved nanocarrier‐based formulations Doxil© and Caelyx© [81]. However, the passive targeting strategy has major limitations as the EPR effect is highly dependent on the tumor's intrinsic biological properties and varies significantly across tumor types, stages, and individuals [82]. For example, metastatic liver cancer and pancreatic cancer exhibit low vascular permeability, resulting in reduced drug accumulation compared with highly vascularized tumors [82, 83]. As a result, nanocarriers relying solely on the EPR effect often face challenges in effectively targeting these tumors, thereby limiting the overall efficacy of therapeutic drugs.

### 3.2 Active Targeting

To enhance the cancer cell targeting capability, active targeting strategies have been developed by incorporating specific targeting moieties, such as antibodies, aptamers, and ligands, onto the surface of nanocarriers [84]. These targeting molecules have high‐affinity interaction with the proteins overexpressed by the tumor cells, including folate receptors, transferrin receptors (TfRs), and epithelial growth factor receptors [84]. Upon reaching the tumor site, targeted nanocarriers interact with cancer cells through specific affinity interactions, facilitating their accumulation within the tumor tissue. Antibodies and antibody fragments, such as antigen‐binding fragments (Fab) and single‐chain variable fragments, play a crucial role in the development of actively targeting nanoparticle systems [85]. These molecules can selectively bind to antigens overexpressed on cancer cells, thereby facilitating enhanced cellular uptake of therapeutic agents and nanoparticle drug delivery systems [86, 87, 88]. Aptamers are single‐stranded oligonucleotides where a library of random oligonucleotide sequences is exposed to the desired targeting ligand. They have emerged as promising active targeting molecules in nanocarrier systems due to their superior cancer‐targeting capability [89]. Aptamers can specifically recognize disease‐associated biomarkers, such as receptors overexpressed on cancer cells, for instance, nucleolin, which is targeted by the AS1411 aptamer [90, 91]. One of the key advantages of aptamers is their high specificity and tunability, allowing precise recognition of target cells while minimizing off‐target interactions [90]. However, a major challenge is their limited in vivo stability, as unmodified aptamers are highly susceptible to nuclease degradation and rapid renal clearance [92]. Ligand‐based targeting strategy utilizes the fact that certain ligand molecules are specifically recognized by receptors that are overexpressed in cancer cells. Various ligand molecules such as hyaluronic acid (HA), FA, and CS have been shown to largely increase the targeting capabilities of nanocarriers [93, 94, 95]. For example, FA‐modified liposomal DOX and paclitaxel formulations have demonstrated preclinical success, shown enhanced tumor accumulation and reduced systemic toxicity [96, 97]. While these studies showed promising results for improved cancer treatment, they did not quantify the ligand density in the nanocarrier formulations. It is necessary to evaluate the optimal ligand concentration in the liposomes to ensure effective clinical translation.

### 3.3 Stimuli‐Responsive Targeting

Stimuli‐responsive nanocarriers represent a transformative approach in targeted drug delivery, engineered to release therapeutic payloads in response to an internal switch associated with disease microenvironments or external triggers [98]. These active delivery systems utilize disease‐related factors such as tumor‐specific pH gradients, elevated glutathione (GSH) levels, overexpressed enzymes or hypoxia, as well as external stimuli like light, magnetic fields, or ultrasound [98]. For example, pH‐sensitive liposomes become destabilized in the acidic tumor microenvironment or within endosomal/lysosomal compartments, thereby enhancing intracellular drug delivery [99]. Similarly, redox‐responsive polymers break down in the high‐GSH cytoplasm, improving the efficacy of encapsulated therapeutics [100]. External triggers, such as near‐infrared light or ultrasound, enable noninvasive control over drug release kinetics from the nanocarriers [101, 102]. Clinical trials have included thermosensitive liposomes (e.g., ThermoDox) in combination with hyperthermia for localized cancer therapy [103]. Despite their promise, stimuli‐responsive nanocarriers face some challenges. The heterogeneity of disease biomarkers, such as varying pH gradients across different tumors, can compromise the reliability of activation [99]. External stimuli like light suffer from limited tissue penetration depth, restricting their utility to superficial or accessible lesions [98]. Additionally, synthesis complexity increases the manufacturing costs of such nanocarriers and raises concerns about long‐term stability [98].

## 4 Applications of Nanocarrier‐Based Targeted Delivery

Targeted delivery using nanocarriers has been widely investigated and applied in the treatment of various diseases, including genetic disorders, cardiovascular conditions, neurodegenerative diseases, and cancers.

### 4.1 Gene Disorders

A particularly promising application is in the treatment of monogenic disorders, where nanocarriers are employed to deliver genetic payloads that can modify or correct disease‐causing genes [104]. For example, lipid nanoparticles functionalized with liver‐targeting ligands such as N‐Acetylgalactosamine (GalNAc) have been used to specifically deliver CRISPR‐based therapies targeting the *ANGPTL3* gene to liver cells in both mouse and nonhuman primate models, offering a potential treatment for homozygous familial hypercholesterolemia [105]. Similarly, multifunctional lipid nanoparticles modified with all‐trans‐retinamine have been shown to deliver plasmid DNA to the retina in the *Rpe65* ^−/−^ mouse model for the treatment of Leber's congenital amaurosis caused by RPE65 gene mutations [106]. In addition, a recent study reported the development of lipid nanoparticles functionalized with β‐d‐galactose‐based ligands to enhance targeted delivery [107]. These nanoparticles were specifically engineered to target the asialoglycoprotein receptor, which is abundantly expressed on the surface of hepatocytes. By facilitating receptor‐mediated endocytosis, this strategy enabled the safe and efficient delivery of mRNA to the liver. The therapeutic mRNA encoded functional Factor VIII (FVIII), aiming to restore protein expression in hemophilia A, a genetic disorder caused by mutations or deficiencies in the F8 gene that results in the absence or dysfunction of FVIII protein [107]. These targeted systems reduce off‐target effects on other healthy tissues and improve therapeutic efficacy.

### 4.2 Neurodegenerative Diseases

Effective targeted delivery across the BBB is critical for treating neurodegenerative diseases [108]. Nanocarriers can be functionalized with targeting molecules such as antibodies to deliver drugs to neurons and glial cells [109]. For instance, polymer nanoparticles coated with polysorbate 80 and loaded with peptide inhibitors of polyglutamine aggregation have been used to treat Huntington's disease in both in vitro and in vivo models [110]. In another study, polymeric nanoparticles modified with rabies virus glycoprotein peptide were reported to successfully cross the BBB and codeliver a therapeutic gene (shRNA) along with epigallocatechin‐3‐gallate to brain tissue in a mouse model of Alzheimer's disease (AD). This dual‐delivery strategy effectively reduced amyloid‐β plaque deposition and inhibited p‐tau‐related fibril formation [111]. Lysosomal storage disorders (LSD), which primarily affect the central nervous system, often cause progressive and severe neurological impairments [112]. To address this, a therapeutic strategy using TfR‐targeted delivery of a plasmid encoding β‐glucuronidase has been developed. Although the resulting enzyme activity was lower than physiological levels, it was still sufficient to provide therapeutic benefit [113]. However, because this strategy depends on systemic intravenous administration, achieving sustained therapeutic benefits for chronic LSD conditions would require repeated dosing at intervals dictated by the duration of transgene expression [113]. Recent advances include the development of stimuli‐responsive nanocarriers that release their therapeutic cargo in response to elevated levels of ROS commonly found in the microenvironment of Parkinson's disease (PD) [114].

### 4.3 Cardiovascular Conditions

In cardiovascular therapy, nanocarriers are used to target atherosclerotic plaques, enhancing localized drug retention [115]. For example, PLGA polymer nanoparticles conjugated with the mZD7349 peptide and loaded with simvastatin have been used to target dysfunctional endothelial cells, showing significantly higher therapeutic efficiency compared with nonconjugated nanoparticles [116]. Lipid nanoparticles modified with a targeting peptide were used to specifically deliver anti‐miR‐712 to the endothelial surface of atherosclerotic lesions overexpressing vascular cell adhesion molecule 1 (VCAM1). In the partial carotid ligation model using *ApoE−/−* mice, this targeted delivery strategy effectively reduced atherosclerosis while minimizing off‐target effects on other tissues [117]. In addition to atherosclerosis treatment, targeted nanocarriers have shown promising potential in myocardial repair and regeneration after infarction. For example, a dual‐targeted lipid‐based complex functionalized with the antimyosin monoclonal antibody 2G4 (mAb 2G4) and the trans‐activator of transcription (TAT) peptide has been developed to deliver therapeutic genes directly to ischemic myocardial tissue, improving localized therapeutic outcomes [118]. Beyond lipid‐based systems, polymeric nanoparticles have also been investigated for targeting acute myocardial infarction (MI). For instance, a peptide–polymer conjugate system was developed using a peptide sequence identified via phage display technology, which demonstrated high affinity for infarcted myocardial tissue. Delivery of this targeted system resulted in improved cardiac function, highlighting its therapeutic promise for post‐MI tissue repair [119].

### 4.4 Cancer Therapy

Cancer therapy remains one of the most intensively investigated applications of targeted nanocarrier systems. By conjugating nanodrugs with ligands that selectively bind to receptors overexpressed on the surface of tumor cells, active targeting can be achieved with high specificity and efficiency [120, 121]. For instance, a HER2‐targeted polymeric drug delivery system was developed using a star‐shaped dendritic polymer conjugated with the topoisomerase I (TOP1) inhibitor SN‐38 and the antigen‐binding fragment of trastuzumab (HER2–Fab). This nanocarrier specifically recognizes and binds to cancer cells overexpressing HER2, resulting in significantly enhanced antitumor efficacy compared with nontargeted counterparts [122]. In recent years, stimuli‐responsive nanoparticles have emerged as a promising strategy for precise tumor targeting [123]. Some functionalized nanoparticles are designed to respond to endogenous tumor microenvironment cues [124]. Functionalized nanoparticles can also be externally activated by exogenous stimuli such as ultrasound, light illumination, or radiation [125, 126, 127]. For example, a lipid–polymer hybrid nanoparticle platform has been developed to enable X‐ray‐induced photodynamic therapy (PDT) specifically targeting human colorectal cancer cells. This multifunctional nanocarrier coencapsulates verteporfin, a clinically approved photosensitizer, and 5‐FU, a widely used chemotherapeutic agent, within a single delivery system. Upon exposure to X‐ray irradiation at 4 Gy, the verteporfin generates ROS, leading to oxidative damage and apoptosis in tumor cells [127]. In cancer vaccines, targeted nanocarriers can enhance the efficiency of immunotherapy by improving antigen delivery and stimulating stronger immune responses [128, 129]. For instance, poly (β‐amino ester)‐based nanoparticles functionalized with T‐cell targeting anti‐CD3e f(ab′)2 fragments were used to specifically deliver CAR‐encoding plasmid DNA to T cells in C57BL/6 mice. This targeted delivery system enhanced T‐cell responses and triggered a robust antitumor effect [130]. Iron oxide nanoparticles modified with mannose have been used to augment antitumor efficacy and enhance neoantigen vaccine through the repolarization of tumor‐associated macrophages and improved coordination of immune cell activity [131].

## 5 Targeting Subcellular Organelles

### 5.1 The Biological Functions of the Main Subcellular Organelles

**FIGURE 1:**
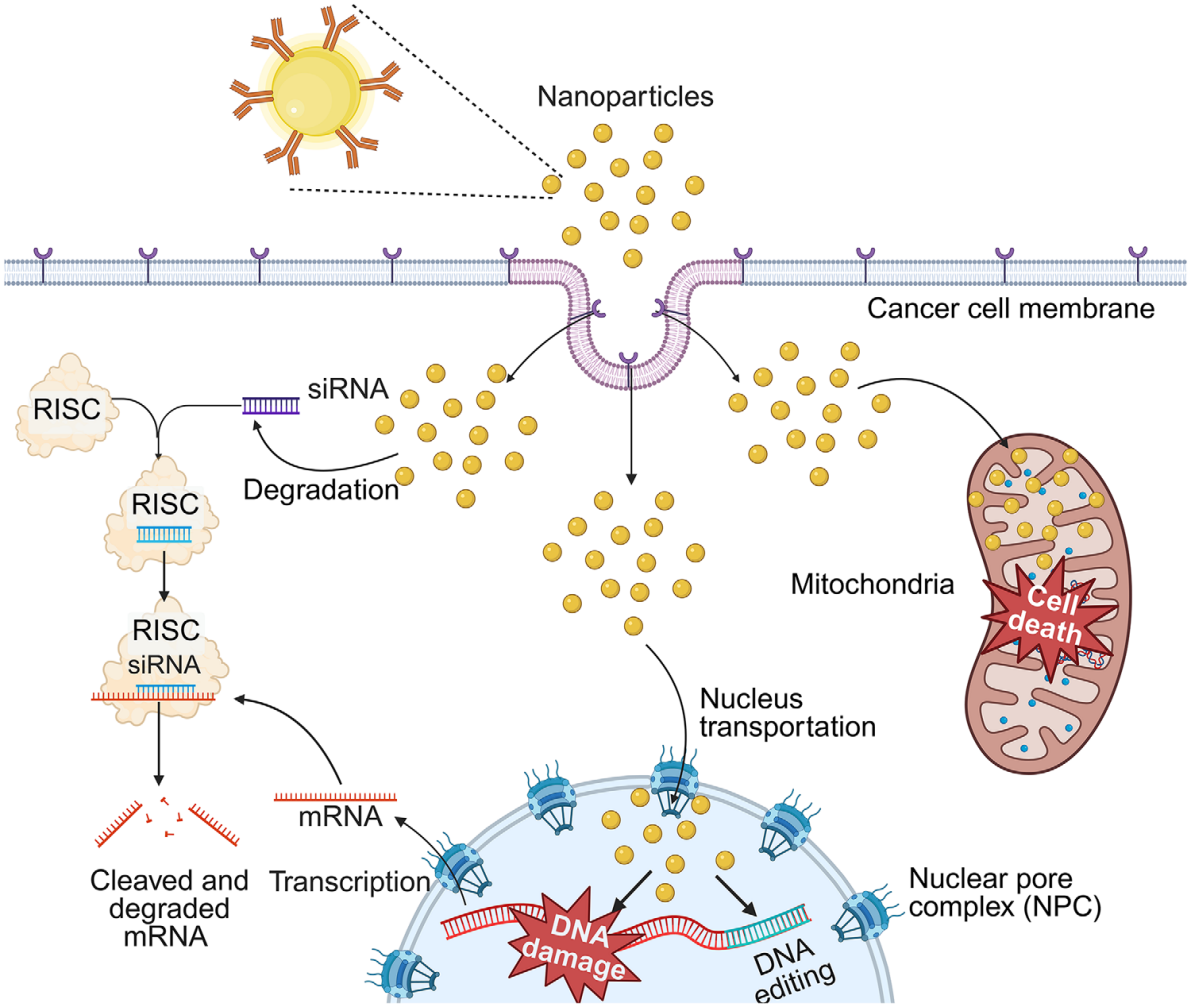
Schematic illustration of intracellular targeting to the cytoplasm, mitochondria, and nucleus. After cellular uptake, nanoparticles deliver genetic cargo into the cytoplasm to mediate gene silencing, target mitochondria to trigger apoptosis, or enter the nucleus through nuclear pore complexes for gene regulation. Created in BioRender. Xu (2025) https://BioRender.com.

Cytoplasm is a semi‐fluid substance within the cell that lies between the cell membrane and the nuclear membrane and fills the entire cell [132]. It is the intracellular portion that contains the organelles, including all structures outside the nucleus [132]. It consists mainly of water, ions, small‐molecule metabolites, proteins, and nucleic acids [132, 133]. The cytoplasm is the main site of several metabolic pathways, including glycolysis, lipid metabolism, and protein synthesis [134]. Furthermore, the cytoplasm serves as the primary site of action for therapeutic miRNA/siRNA agents, allowing them to exert their effects and ultimately maximize therapeutic outcomes [135]. Once siRNA is taken up by cells, it is recognized by the RNA‐induced silencing complex (RISC). This complex uses the siRNA as a guide to find and bind to specific mRNA molecules that are complementary to the siRNA sequence [136, 137]. By doing so, the RISC–siRNA complex can cut and destroy these mRNA molecules, preventing them from being translated into proteins. This process effectively reduces the expression of genes that are involved in disease development (Figure 1) [138, 139]. Presently, a broad range of viral and nonviral vectors has been engineered to enhance the escape of siRNA from endosomes. This allows for the release of siRNA into the cytoplasm, ultimately increasing the effectiveness of gene silencing [140, 141].

Mitochondria are oval or oblong cylindrical organelles consisting of two membrane systems [142]. Unlike other organelles, mitochondria have their DNA called mitochondrial DNA (mtDNA), which mainly encodes several proteins related to the function of mitochondria [143]. The primary role of the mitochondria is to facilitate cellular respiration and generate adenosine triphosphate, which is the key energy molecule within the cell [143, 144]. It also plays an important role in the regulation of cellular function. For instance, mitochondria have been found to be suitable subcellular targets for ROS generated by PDT (Figure 1) [145, 146]. The ROS‐induced effects are capable of disrupting cellular functions such as proliferation [147, 148], while mtDNA damage initiates diverse cell death mechanisms [149, 150]. Consequently, mitochondria‐targeted PDT has proven to be more effective than a nontargeted alternative, allowing greater specificity and potentially smaller effective drug doses. In addition, mitochondrial dysfunction plays a critical role in the pathogenesis of PD and AD, targeting mitochondrial dysfunction could be efficiently implicated for PD and AD treatment [151, 152].

The nucleus is a vital organelle that houses the cell's genetic material and governs the cell's growth, development, and metabolism [132]. Positioned typically at the center of the cell, the nucleus comprises a nuclear membrane and a nucleolus. It serves as the primary repository for the cell's DNA, which contains the genetic instructions necessary for the functions of an organism [132]. Chemo‐drugs can interact with nuclear DNA through various mechanisms, such as DNA crosslinking and topoisomerase inhibition [153]. These interactions disrupt normal DNA structure and functions, leading to DNA damage and ultimately affecting cellular processes and viability (Figure 1). Additionally, compared with other strategies that target different subcellular areas or have a random distribution, nucleus‐targeted PDT/photothermal therapy (PTT) is a more direct and efficient approach to kill cancer cells using light or heat [154, 155, 156]. In addition to chemotherapy and PDT/PTT delivery, it is also crucial to efficiently deliver genetic materials into the cell nucleus. This is particularly important for utilizing CRISPR/Cas gene editing tools, which hold promise for treating cancer and genetic disorders [157, 158]. To ensure successful gene editing, both the Cas9 protein and the sgRNA need to be transported into the nucleus of target cells [159, 160]. Once inside the nucleus, they can initiate the gene editing process by targeting and modifying specific DNA sequences [159]. However, this technology may have unintended off‐target and cytotoxic effects, especially when administered systemically [161, 162]. Therefore, effective nuclear targeting is essential to ensure optimal delivery and expression of therapeutic genes.

Precise targeting of the cytoplasm, mitochondria, and nucleus plays a pivotal role in drug development for disease treatment. Such targeted approaches not only enhance treatment specificity and efficacy but also minimize off‐target effects and associated side effects.

### 5.2 Strategies on Nuclear‐Targeted Delivery via Nanocarriers

#### 5.2.1 Nuclear Pore Complex: Regulator of Nucleocytoplasmic Transport

The nuclear pore complex (NPC) is a proteinaceous structure responsible for controlling the movement of various molecules, including proteins, RNA molecules, and signaling molecules, between the nucleus and the cytoplasm [163, 164, 165]. It is composed of nucleoporins, a specific class of proteins that come together to construct a channel‐like structure with a central pore [166, 167]. Ions and small molecules can passively diffuse through a pore. However, larger molecules, typically those exceeding 40–60 kDa in size, require the assistance of nuclear transport receptors [168]. These receptors recognize specific signals called nuclear localization sequences (NLS) or nuclear export sequences on the molecules, allowing them to bind and facilitate their transport into or out of the nucleus [169, 170]. This process ensures that only the appropriate molecules are able to enter or exit the nucleus, maintaining cellular integrity and function.

#### 5.2.2 Strategies for Nucleus Transportation

**FIGURE 2:**
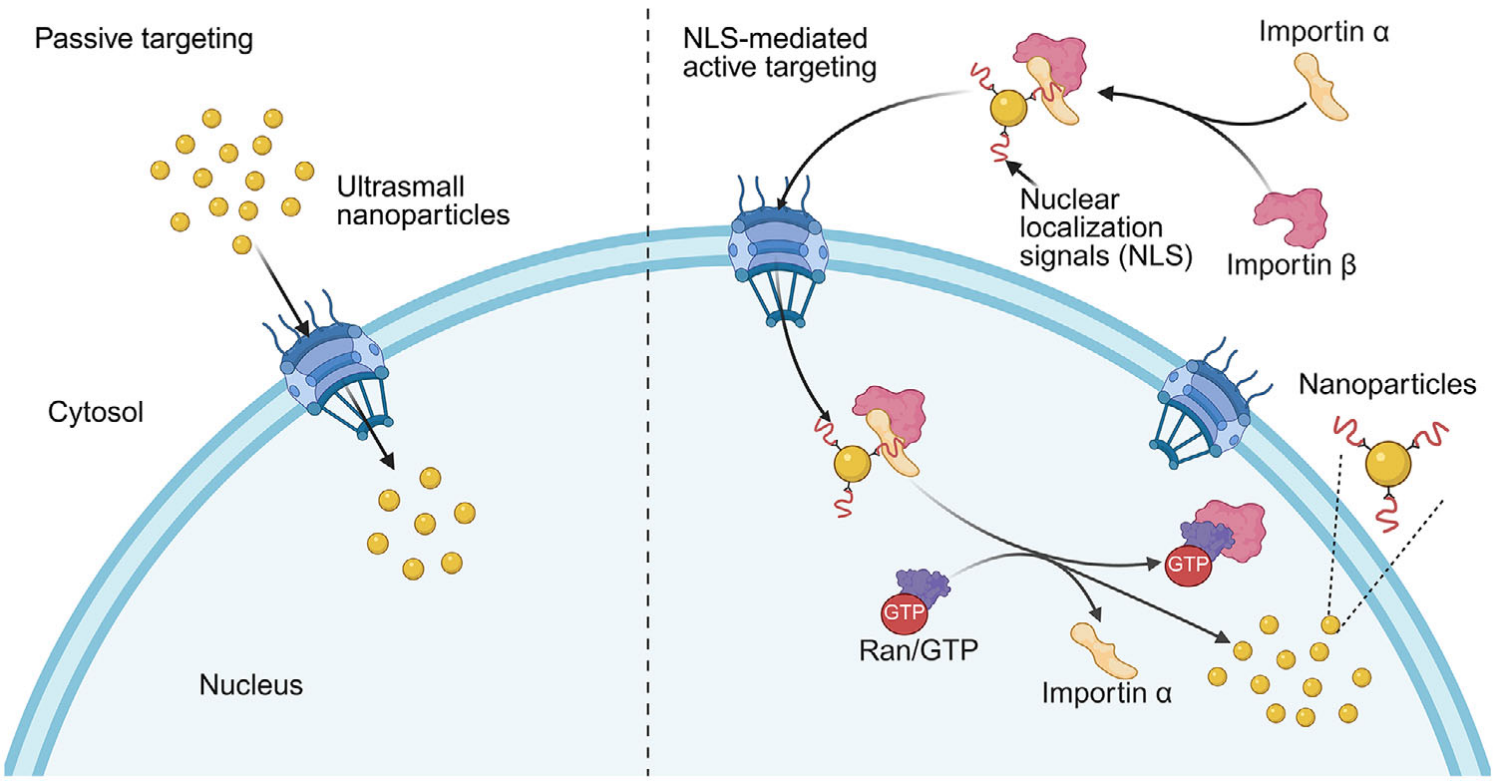
Passive and nuclear localization sequence (NLS)‐mediated active nuclear transport of nanoparticles. In passive transport (left), ultrasmall nanoparticles diffuse through nuclear pore complexes. In active transport (right), NLS‐modified nanoparticles bind to importin α/β and are carried into the nucleus, where Ras‐related nuclear protein (Ran)‐GTP mediates cargo release. Created in BioRender. Xu (2025) https://BioRender.com.

Synthetic nanocarriers, in principle, need to transport drugs or genetic materials to the nucleus to exert functions for the treatment of nucleus‐related diseases, including cancers and genetic disorders. There are intracellular barriers for these delivery vehicles to accumulate in the nucleus during the transportation. One major intracellular challenge faced by nanocarriers is their entrapment within the endo/lysosomal compartments after being engulfed by the cell [171]. Endosomes and lysosomes are membrane‐bound compartments within the cell that are involved in the degradation and recycling of cellular components [10, 172]. When nanocarriers are taken up by the cell through endocytosis, they often get trapped within these compartments, leading to their degradation and limited access to the target site, such as the nucleus. Another obstacle to successful nuclear transport is the requirement for drugs or genes to navigate through the cytosol, which is a densely packed and molecularly crowded environment at the molecular level [173]. This means that there are many molecules and cellular components present in the cytosol, making it difficult for drugs or genes to reach the nucleus. To overcome these challenges, researchers have focused on engineering nanocarriers, which can be categorized into two types, passive and active targeting (Figure 2).

##### 5.2.2.1 Passive Targeting

The nucleus is enclosed by a double nuclear membrane, which limits direct interaction between the nucleus and the cytoplasm. However, it is not entirely isolated as NPCs embedded in the nuclear membrane facilitate material exchange and information transfer [174]. Passive nuclear transport is an energy‐independent process driven by Brownian motion and the intrinsic affinity of molecules for nuclear components [175, 176]. Through this mechanism, small hydrophilic molecules can diffuse across NPCs into the nucleus by following concentration gradients [177].

Passive targeting involves the design of nanocarriers that can rely on the unique properties of the nucleus (e.g., size and density) to facilitate drug or gene entry into the nucleus [173]. This approach takes advantage of nanocarrier characteristics such as size, shape, and surface charge to passively accumulate at the nucleus. Ultrasmall (<10 nm) nanoparticles have received particular attention for passive targeting due to their ultra‐small size. In a study, researchers utilized tiopronin‐coated AuNPs for passive targeting of cell nuclei [178]. Tiopronin was used to prevent the coagulation of AuNPs, ensuring their stability in physiological environments. The study found that only AuNPs smaller than 10 nm (2 and 6 nm) were able to enter the nucleus, while larger ones (10 and 16 nm) remained in the cytoplasm. The researchers also used 2 nm AuNPs as a carrier to directly deliver triplex‐forming oligonucleotides (TFOs) into the nucleus. The results demonstrated that compared with unmodified TFOs, the conjugation of TFOs with 2 nm AuNPs significantly improved the efficiency of TFOs entering the nucleus, enhancing the knockdown efficacy. Another study revealed that 2 nm AuNPs were able to penetrate the nucleus, which is a crucial step for drug delivery targeting the nucleus. Furthermore, this study demonstrated that these AuNPs are capable of crossing the BBB, which is a highly selective and protective barrier surrounding the brain [179]. This finding may offer possibilities for utilizing AuNPs in the treatment of brain‐related diseases and disorders. Recently, researchers have developed small MOF nanocarriers that specifically target the nucleus for treating orthotopic pancreatic carcinoma using type I sonodynamic treatment (SDT) [180]. This MOF nanocarrier is loaded with Ti‐tetrakis (4‐carboxyphenyl) porphyrin, a photosensitizer. Due to its small size (<10 nm) and charge reversal property, the MOF nanocarrier effectively enters the cells and directly targets the nuclei. This targeted approach enhances the efficiency of SDT, making it a more effective treatment option. Despite ultrasmall nanoparticles having demonstrated excellent nuclear‐targeting capabilities, they face challenges in terms of stability and industrial manufacturing [181]. Another concern is their lack of gradability and biocompatibility, which causes high tissue toxicity [182, 183]. Shape and surface charge are also critical parameters influencing the nuclear uptake of nanocomposites. Hinde et al. [184] studies on the behavior of nanoparticles with various shapes at the subcellular level. The authors reported that nanoparticles with a high aspect ratio (length‐to‐width ratio), such as rod‐shaped and worm‐like nanoparticles, exhibit higher accumulation in the nucleus compared with micelle and vesicle nanoparticles. When encapsulating DOX, rods and worms delivered substantially more DOX to the nucleus compared with spherical nanoparticles. This could be attributed to the fact that nanoparticles with high aspect ratios (such as worms and rods) exhibit higher efficiency in passive diffusion into the nucleus compared with spherical nanoparticles [184]. The nuclear membrane is negatively charged, allowing positively charged nanoparticles to bind to the negatively charged nuclear membrane, thereby facilitating their entry into the nucleus [185, 186]. Lee et al. [187] designed positively charged quantum dots capable of directly labeling the nucleus in both fixed and live HeLa cells. In contrast, negatively charged quantum dots were confined to the cytoplasm. These findings highlight that the charge of nanoparticles is a critical factor influencing their intracellular distribution, including nuclear localization [187].

The passive targeting strategy is largely dependent on the size, shape, and surface charge of nanoparticles, which constrains its versatility and broader applicability.

##### 5.2.2.2 NLS‐Mediated Active Targeting

The active targeting strategies can be achieved by modification of the NPC itself [188] or by surface modification of nanocarriers with nuclear‐targeting ligands or peptides [189]. In this section, we primarily focus on nanoparticle engineering to enhance nuclear targeting efficiency. Active targeting involves the use of specific ligands or peptides that can recognize and bind to components within the nucleus. This recognition and binding process leads to the accumulation of therapeutic molecules specifically within the nucleus. In contrast to passive targeting, which is limited by the physical properties of freely diffusing drug molecules or nanoparticles, active targeting can improve transport efficiency from the plasma membrane to the nucleus and increase the macromolecules or nanoparticles to enter the nucleus efficacy [190, 191]. These peptides have binding affinities for nuclear transport receptors located in the cell nucleus. By attaching these peptides to the surface of drugs or nanocarriers, they can effectively deliver the drugs or gene editing components into the nucleus for precise therapy. NLS are generally short peptides with signal sequences that direct proteins to the nucleus [192]. Extensive research on NLS has resulted in significant progress, enabling successful targeting of various small molecules, proteins, and nucleic acids to the nucleus [193]. NLS can be categorized into two main types: classical NLS (cNLS) and nonclassical NLS (ncNLS) [192]. The cNLS typically consists of positively charged amino acids, such as lysine and arginine [194]. It facilitates entry into the cell nucleus by binding to nuclear transport receptors like importin. This signal is generally recognized and transported by the primary nuclear transport machinery, playing an important role in processes such as cell division and stress response [192, 195]. Within cNLS, there are two subtypes: monopartite (MP) and bipartite (BP). MP NLS is composed of a cluster of 4–8 basic amino acids, typically containing four or more positively charged residues [192]. Its characteristic motif requires consecutive lysine residues at the N‐terminus, forming a loose consensus sequence of K(K/R)X(K/R). Only these positively charged sequences are functional in facilitating nuclear transport [196]. BP NLS is composed of two clusters of 2–3 positively charged amino acids, separated by a linker region of 9–12 amino acids [197]. These upstream and downstream clusters are interdependent and play a critical role in enabling nuclear transport. The characteristic motif of BP NLS can be represented as R/K(X)10‐12KRXK [198]. The ncNLS is a nonspecific combination of amino acids that is not necessarily rich in positively charged residues. Its localization mechanism often depends on the protein's folding state or specific structural features [199, 200]. Unlike cNLS, it utilizes diverse transport mechanisms and can achieve nuclear transport via complexes that rely on signaling molecules [200, 201]. This flexibility enables ncNLS to adapt its function in response to changes in the cellular environment and physiological state [202].

NLS are recognized by the karyopherin importin α/β, forming a complex that interacts with NPC to facilitate translocation through the nuclear pore (Table 1).

**TABLE 1:** Nuclear localization signal (NLS) sequences used for nuclear target.

Category	Type	NLS amino acid sequence	Transport protein	Protein source	References
Classical nuclear localization signal (cNLS)	Monopartite NLS	PKLKRQ	Importin α/β1	VACM‐1	[203, 204]
		RPRK	Importin α/β1	CXCR4	[205, 206]
		RRARRPRG	Importin α/β1	VP1	[207, 208]
		PAAKRVKLD	Importin α/β1	C‐MYC	[209, 210]
		VSRKRPRP	Importin α/β1	T‐Antigen	[211, 212]
		QRKRQK/EEKRKR	Importin α3/α4	NF‐κB	[213]
		PKKKRKV	Importin α	SV40	[214, 215]
	Bipartite NLS	KRPAATKKAGOAKKKK	Importin α/β1	Nucleoplasmin	[216]
		KGKKGRTQKEKKAARARSKGKN	Importin α/β1	ING4	[217, 218]
		RKRCAAGVGGGPAGCPAPGSTPLKKPRR	Importin α/β1	IER5	[219]
		RKPVTAQERQREREEKRRRRQERAKEREKRRQERER	Importin 7	ERK5	[220, 221]
Nonclassical nuclear localization signals (ncNLS)	Proline tyrosine NLS	RSGGNHRRNGRGGRGGYNRRNNGYHPY	Importin β2	HRP1	[222]
		TLLLRETMNNLGVSDHAVLSRKTPQPY	Importin β2	UL79	[223]
		FGPGKMDSRGEHRQDRRERPY	Importin β2	FUS	[224, 225]
		NNOSSNFGPMKGGNFGGRSSGPYG	Importin β2	hnRNPA1	[226]
		NQQPSNYGPMKSGNFGGSRNMGGPYG	Importin β2	hnRNPA2/B1	[227]
	Other ncNLS	SANKVTKNKSNSSPYLNKRKGKPGPDS	Importin β & RanBP5/7	Pho4	[228]
Other	Multiple NLS	RNKKKKRKVIK	Importin α/β1	NLS‐RARα	[229]

**FIGURE 3:**
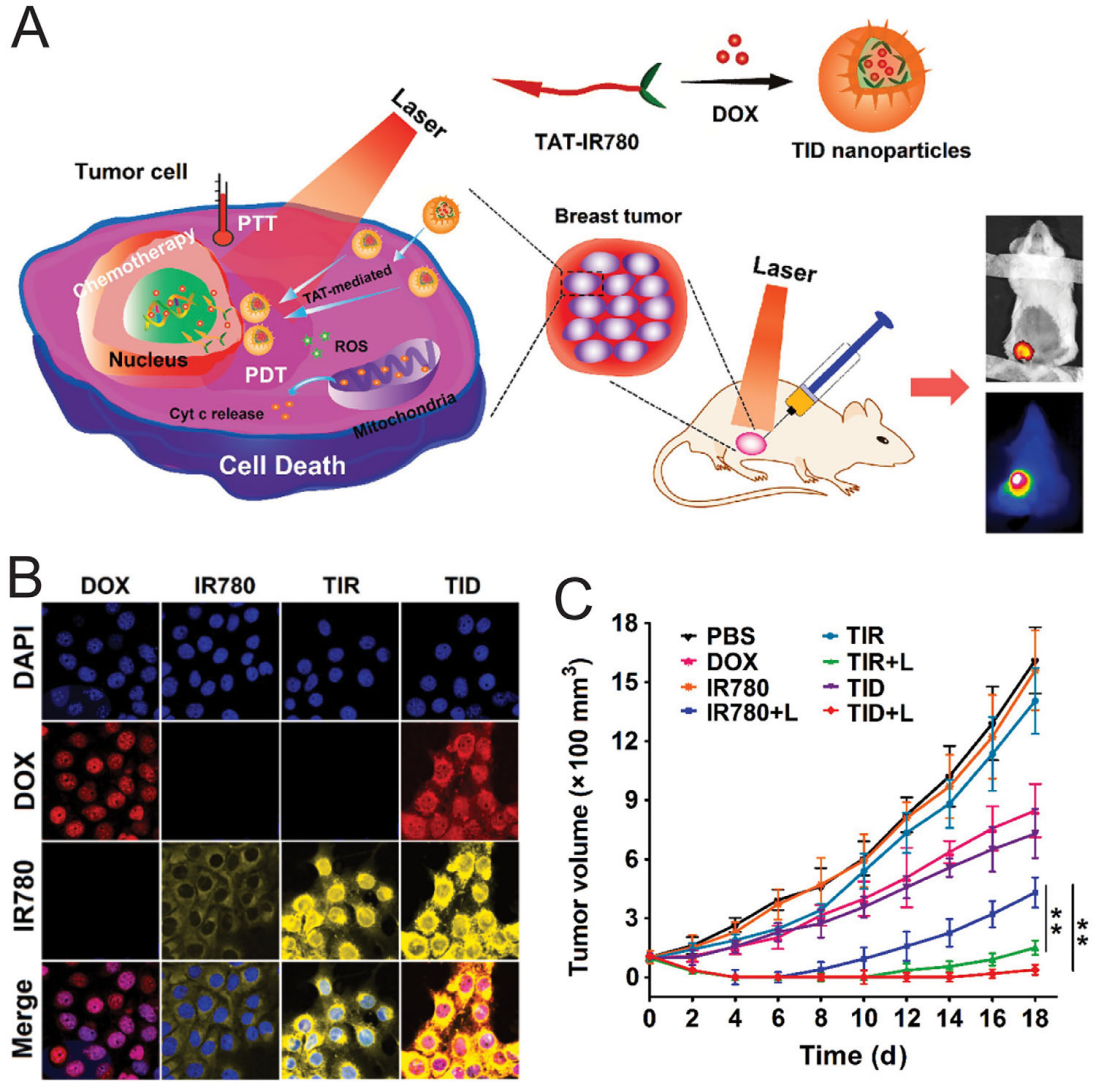
Nucleus‐targeted photothermal/photodynamic therapy using trans‐activator of transcription (TAT)‐modified nanoparticles. (A) Illustration of TAT–IR780 (TIR), TAT–IR780–DOX (TID) nanoparticle‐mediated photothermal therapy/photodynamic therapy. (B) Confocal images of 4T1 cells treated with targeted nanoparticles (TIR and TID) and other free molecules after 6 h. (C) Tumor change after the treatment with free IR780, TIR, and TID nanoparticles with/without light trigger. Reproduced with permission [156]. Copyright 2019 Elsevier B.V.

This process is facilitated through the hydrolysis of guanosine triphosphate (GTP) [192, 230]. As a result, various molecules can freely transport to the nucleus. The use of NLS peptides for nanoparticle modification is a well‐established strategy in the field of nuclear targeting. One of the well‐known NLS peptides is the TAT. The original TAT peptide consists of 11‐amino acids (YGRKKRRQRRR) and contains a high positive charge, which is essential for its nuclear targeting ability [231, 232]. The positively charged residues allow the TAT peptide to interact with negatively charged components of the cellular membrane, facilitating its uptake into cells [192, 233]. Once inside the cell, the TAT peptide binds to importin proteins, which are responsible for transporting molecules into the nucleus [234]. This interaction allows the TAT peptide, along with the larger nanoparticles it is conjugated with, to be efficiently delivered into the nucleus [235, 236]. In a study conducted by Wan et al. [156] developed the self‐assembled nanoparticles (TID) by conjugating a photosensitizer, IR780 with TAT peptide. These targeted nanoparticles were then assembled with the chemotherapeutic drug DOX (Figure 3A). Mediated by the TAT peptide, these nanoparticles exhibited enhanced nuclear targeting capability in breast cancer cells compared with free IR780 molecules and DOX, as shown in Figure 3B. Upon laser irradiation, the nanoparticles exhibited dual therapeutic effects of PTT and PDT. The PDT/PTT effects were achieved by damaging the genetic material within the cell nucleus and generating cytotoxic ROS, thereby enhancing the therapeutic efficacy of the targeted nanoparticles compared with pure IR780 or DOX molecules (Figure 3C).

NLS peptides can also be incorporated into nonviral gene carriers, such as polymer and liposomes, aiming to enhance the delivery of plasmid DNA specifically to the nucleus, improving transfection efficiency and therapeutic efficacy. In a study, CS/DNA complexes that are directly incorporated with NLS peptide (KPKKKRKV) efficiently deliver pEGFP‐C2 plasmid DNA to Hela cells [237]. The highest transfection efficiency was significantly observed in the CS/DNA complex at the weight ratio of 8 with the addition of 120 µg NLS, which was 74‐fold higher than that in the cells transfected with the CS/DNA complex alone. In another study, Rosada et al. [238] employed a combination of a synthetic NLS peptide (KCRGKVPGKYGKGPKKKRKVC‐amide) and a cationic liposome to deliver a plasmid DNA encoding the 65‐kDa heat shock protein gene (DNAhsp65) for the treatment of tuberculosis (TB). The cationic liposome utilized in this study consisted of lipids such as 1,2‐dioleoyl‐3‐trimethylammonium‐propane, 1,2‐dioleoyl‐sn‐glycero‐3‐phosphoethanolamine, and l‐α‐phosphatidylcholine. This liposome formulation aimed to provide sustained protection against TB while reducing the required dose of DNA. The NLS‐mediated liposomes demonstrated superior therapeutic effects against TB compared with the cationic liposome/DNA hsp65 gene vaccine and were similar to the naked DNA treatment. These findings suggest that delivering DNA to the cell nucleus via NLS activity can enhance gene transcription, leading to an effective immune response against the relevant pathogen.

**FIGURE 4:**
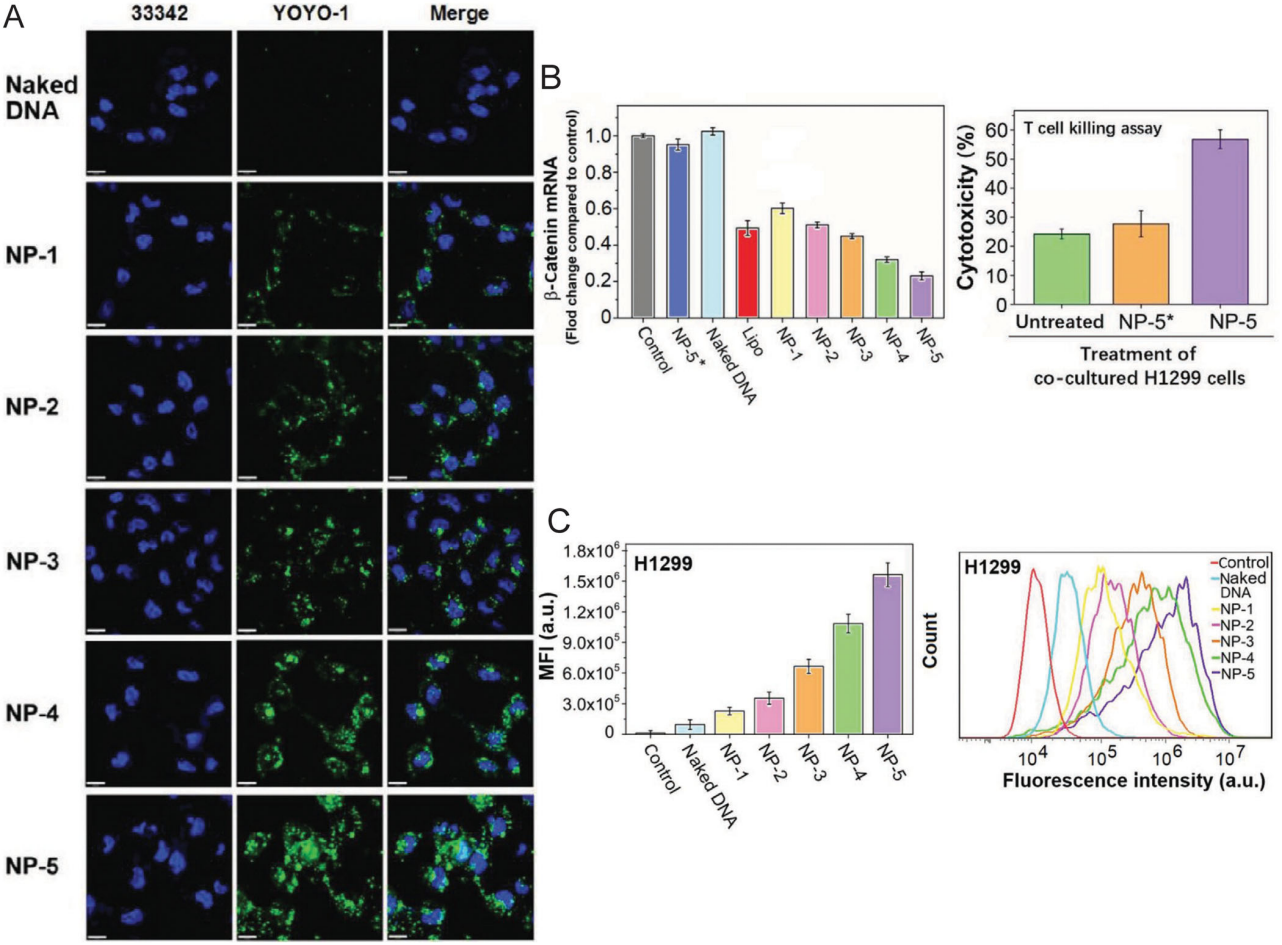
Enhanced nuclear delivery and immunomodulation using dual‐targeted nanocarriers. (A) Confocal images for the nanocarrier, nucleus, and overlap. NP‐1: PS/CaCO3/Cas9 plasmid, NP‐2: HA/PS/CaCO3/CRISPR‐Cas9 plasmid, NP‐3: PHA/HA/PS/CaCO3/Cas9 plasmid, NP‐4: AHA/HA/PS/CaCO3/Cas9 plasmid and NP‐5: AHA/PHA/PS/CaCO3/Cas9 plasmid. (B) Wnt/β‐catenin and activated T cells cytotoxic changes. (C) Comparison of programmed death‐ligand 1 (PD‐L1) expression in untreated cells (control) and NP‐5. Reproduced with permission [91]. Copyright 2020 WILEY‐VCH Verlag GmbH & Co. KGaA, Weinheim.

In a recent report, He et al. [91] fabricated an AS1411/TAT‐functionalized polymer nano‐system that can effectively deliver CRISPR–Cas9 plasmid for β‐catenin knockout to reverse tumor immunosuppression. This delivery vector was modified with two types of HA conjugates, one with an aptamer, AS1411 for tumor cell/nuclear targeting, and the other with a TAT–NLS peptide for cell penetration and nuclear translocation. By combining these two functionalities, this delivery system can specifically deliver the genome‐editing plasmid to tumor cell nuclei. As shown in Figure 4A, the targeted nanoparticles modified with TAT–NLS (NP‐3) and AS1411 (NP‐4) exhibited higher nuclear‐targeting efficacy compared with the nontargeted nanoparticles (NP‐1 and NP‐2). The combination of TAT–NLS and AS1411 modifications (NP‐5) achieved the highest nuclear targeting efficiency. As a result, the Wnt/β‐catenin pathway is inhibited (Figure 4B), leading to the suppression of tumor cell growth. Additionally, this downregulates PD‐L1 expression (Figure 4C), restoring the antitumor activity of T cells (Figure 4B).

The active targeting strategy allows for the engineering of nanoparticles beyond their physical properties by conjugating them with nuclear‐targeting molecules, thereby improving their targeting specificity and therapeutic efficacy.

## 6 Current Challenges in Nanocarrier‐Based Targeted Delivery

Despite extensive research on nuclear targeting, some obstacles still exist in achieving efficient delivery of therapeutic cargo to the nucleus.

### 6.1 Complex Physiological Environment

One major obstacle is primarily related to the complex physiological environment and intricate biochemical processes involved. To ensure sufficient nuclear accumulation, it is critical for nanocarriers to have adequate stability within the physiological environment, avoiding undesired interactions with the surrounding media from the systemic circulation [239]. Once the nanocarriers reach the blood vessels near the target sites, they encounter an additional challenge known as the epithelial tissue before entering the targeted sites. For instance, a major hurdle arises when the size of the particles exceeds 5 nm in diameter, as they face difficulties in crossing the capillary epithelial barrier [240]. Once the nanocarriers have passed through the epithelial layer, they begin to attach to the cell membrane of the target cells and are subsequently internalized into the cytosol, primarily through the cellular endocytosis pathway [241, 242]. This process can vary depending on the cell type, resulting in different intracellular processes [243]. The diversity of endocytosis pathways can result in varying efficiencies of subcellular delivery, including targeting the nucleus, even when using the same delivery system. Endocytic processes can also lead to the internalization of the nanocarriers into endosomes or lysosomes [244], preventing its nuclear delivery. For instance, the nanocarriers can become trapped within endo/lysosomal compartments. These compartments are part of the cellular process responsible for breaking down cellular components. Even when nanocarriers are modified with nuclear‐targeting molecules, this does not always translate into improved functional outcomes. For example, an NLS derived from the transcription factor TFIIE‐β (SKKKKTKVC) significantly enhanced nuclear uptake but failed to increase antisense activity [10, 37]. This observation highlighted that while nuclear targeting is an important step, endosomal escape remains a major barrier to achieving effective intracellular delivery and functional gene modulation [245]. To ensure therapeutic agents successfully reach the nucleus and exert their intended effects, it is crucial for the delivery system to escape from endo/lysosomal compartments promptly and avoid enzymatic degradation within lysosomes [10, 37].

### 6.2 Off‐Target Effect of Certain Targeting Molecules

Another concern related to NLS‐mediated nuclear targeting is the potential off‐target effect of certain NLS, such as TAT peptides, which may impede achieving the precise nuclear targeting. It may result in the accumulation of cargo in organelles other than the nucleus, such as mitochondria [246, 247]. This prevents the cargo from reaching its intended nuclear destination. Additionally, other off‐target effects associated with the nanocarrier itself can disrupt normal cellular processes, potentially leading to cellular dysfunction or toxicity [248]. Therefore, it is vital to minimize off‐target effects when designing strategies for nuclear targets. This will ensure the efficient and specific delivery of cargo to the intended nuclear compartment.

### 6.3 Stability and Toxicity Issues of Nanocarriers

Other targeted nanocarriers also face significant challenges related to toxicity and stability. These challenges not only affect the therapeutic efficacy but also pose significant hurdles for clinical translation. Due to their unique physicochemical properties, nanocarriers can interact with biological systems in unpredictable ways, raising toxicological concerns such as nonspecific protein binding and organ accumulation. The high surface reactivity of nanocarriers can result in the adsorption of plasma proteins, which may trigger an immune response and subsequent inflammation [249]. This immune activation can lead to systemic toxicity if not properly controlled. Stability is a critical factor influencing the in vivo performance of nanocarriers. Interactions between the nanocarriers and organs can lead to aggregation or premature degradation of the nanoparticles, reducing their circulation time and altering their biodistribution [250, 251]. This not only limits therapeutic efficacy but also increases the risk of systemic side effects.

### 6.4 Manufacturing Complexity of Targeted Nanoparticles

In addition to challenges encountered at the preclinical stage, the complexity of nanoparticle manufacturing presents significant challenges in achieving reproducibility, maintaining batch‐to‐batch consistency and meeting established quality benchmarks, particularly for targeting technologies that are technically intricate or difficult to replicate. For example, lipid‐based nanoparticles are highly sensitive to manufacturing and storage conditions. Their triglyceride components can undergo polymorphic transitions from the metastable α‐form to the more thermodynamically stable β‐form, leading to crystalline aggregation. This reduces the amorphous regions within the lipid matrix and may result in premature drug leakage [252, 253]. Furthermore, nanocarriers are highly susceptible to both physical and chemical changes during storage, which presents substantial challenges for large‐scale manufacturing. Oxidative degradation, in particular, can alter surface charge, drug release profiles, and particle stability, while potentially generating toxic byproducts that reduce therapeutic efficacy [254, 255].

Nanocarrier‐based therapeutics are regulated by agencies such as the United States Food and Drug Administration and the European Medicines Agency, which require these systems to meet strict standards of quality, safety, and efficacy prior to approval [256]. To obtain regulatory authorization, manufacturers must comply with established quality guidelines, conduct comprehensive characterization and toxicology studies, and fulfil specific regulatory requirements for clinical trials.

## 7 Future Directions and Conclusions

Some promising areas in nanocarrier research include personalized nanomedicine and the development of technologies that integrate diagnostics and therapeutics. Nanoparticle‐enabled medicine offers excellent cargo flexibility, making it ideal for personalized treatments [257, 258]. For example, in cancer therapy, individual biomarkers and genomic mutations play a crucial role in guiding clinical decisions. Nanocarriers can be tailored to incorporate patient‐specific molecular information, enabling personalized nanomedicine that precisely target individual patients [41]. This approach represents a significant advancement over conventional chemotherapy, which is administered nonspecifically and often yields limited therapeutic efficacy across diverse patient populations. Additionally, the integration of smart technology with drug delivery systems enables real‐time monitoring, allowing for treatment adjustments and leading to improved therapeutic outcomes [259].

In addition, the integration of artificial intelligence (AI) and computational modeling holds great promise for optimizing the design and development of nanocarrier‐based formulations, particularly those involving lipid nanoparticles. For instance, the classical four‐component lipid nanoparticle formulation framework can theoretically yield over 10^10^ possible combinations, making empirical screening both time‐consuming and cost‐prohibitive [260]. To overcome this challenge, data from experimental formulations have been used to train predictive models, allowing for the virtual screening of large lipid libraries, such as one comprising over 40,000 candidate lipids [261]. One example is Mana.bio, an AI‐based drug delivery startup that has developed a platform that combines AI with lipid nanoparticle formulation, significantly accelerating the discovery of high‐performing candidates. AI‐based machine learning models have also been applied to predict delivery efficiency, enabling the rapid and systematic development of ionizable lipids with minimal experimental effort. For example, Lewis et al. [262] constructed a library of ionizable lipids and used a trained machine learning model to predict transfection efficiency, identifying lipid tail length as the most influential chemical feature associated with delivery performance. Similarly, Li et al. [263] trained a machine learning model to screen a library of 40,000 ionizable lipid candidates. The lipid nanoparticles formulated with the selected top‐performing lipids demonstrated improved in vitro transfection efficiency and reduced off‐target delivery [263]. Furthermore, machine learning algorithms can accelerate the clinical translation of nanomedicines by predicting the optimal physicochemical properties tailored to specific cancer subtypes. These tools offer a rational design pathway for engineering nanocarriers that align with patient‐specific tumor biology and therapeutic needs, thereby supporting the development of targeted delivery systems for personalized nanomedicine [264].

In summary, targeted nanocarriers hold great potential for advancing precise medicine strategies and enhancing therapeutic efficacy. Although significant progress has been made in preclinical research, further advancements in their industrial development and clinical translation are necessary to ensure the safe and efficient implementation of precise nanomedicine. These advancements are crucial in addressing the challenges of drug resistance and limited therapeutic effects. Continued research and development efforts in this area will contribute to the advancement of precise nanomedicine and its potential to enhance patients’ quality of life.

## Author Contributions

Z. Xu conducted the literature search, drafted the manuscript, and designed the figures. Y. Xie and W. Chen contributed to editing the content and providing the figures. W. Deng contributed to the conceptualization of the study and manuscript review and editing. All authors have read and approved the final manuscript.

## Ethics Statement

The authors have nothing to report.

## Conflicts of Interest

The authors declare no conflicts of interest.

## Data Availability

The authors have nothing to report.
